# Rapid Detection and Quantification of DMNB Vapors Using a Handheld Ion Mobility Spectrometer Operated near Ambient Temperature

**DOI:** 10.3390/s26072047

**Published:** 2026-03-25

**Authors:** Victor Bocoș-Bințințan, Tomáš Rozsypal, Alin-Gabriel Moraru, Maria-Paula Bocoș-Bințințan, Adrian Pătruț, Petrișor Pătrașcu

**Affiliations:** 1 Faculty of Environmental Science & Engineering, Babeș-Bolyai University, Str. Fântânele nr. 30, 400294 Cluj-Napoca, Romania; alingabriel.moraru17@gmail.com; 2 SC Transcend SRL, Str. Arinilor nr. 13, 400568 Cluj-Napoca, Romania; 3 Nuclear, Biological and Chemical Defence Institute, University of Defence, Vita Nejedleho 1, 68201 Vyskov, Czech Republic; 4 Faculty of Veterinary Medicine, University of Agricultural Sciences and Veterinary Medicine, Calea Mănăştur 3-5, 400372 Cluj-Napoca, Romania; bocos.maria@gmail.com; 5 Faculty of Chemistry & Chemical Engineering, Babeș-Bolyai University, Str. Arany János nr. 11, 400028 Cluj-Napoca, Romania; apatrut@gmail.com; 6 Raluca Ripan Institute for Research in Chemistry, Babeș-Bolyai University, Str. Fântânele nr. 30, 400294 Cluj-Napoca, Romania; 7 Strategic Department, “Carol I” National Defence University, Șos. Panduri nr. 68-72, 050662 Bucharest, Romania; patrascupetrisor@yahoo.com

**Keywords:** ion mobility spectrometry, 2,3-dimethyl-2,3-dinitrobutane (DMNB), ICAO markers, plastic explosives, trace detection

## Abstract

**Highlights:**

**What are the main findings?**

- Quantification of DMNB was done using test atmospheres generated from neat vapor sources using dynamic methods; calibration from low ppb_v_ to several ppm_v_ has been performed.
- Vapors of DMNB simultaneously produced both positive and negative IMS spectra, each including only one product ion peak; sensitivity in the positive ion mode is about three times higher than in the negative ion mode.

**What are the implications of the main findings?**

- Fast detection of main marker for plastic explosives DMNB (2,3-dimethyl-2,3-dinitrobutane) was achieved by IMS at ambient temperature, with limits of detection and quantification in the low ppb_v_ range.
- These findings can be applied in real-world scenarios for indirect sensing of plastic explosives.

**Abstract:**

The detection of plastic explosives in vapor form is extremely challenging due to the very low volatility of their primary components, such as RDX and PETN. To overcome this limitation, volatile chemical markers like 2,3-dimethyl-2,3-dinitrobutane (DMNB) are added to explosive formulations to enable indirect vapor detection. This study presents a rapid method for detecting and quantifying DMNB vapors using a handheld ion mobility spectrometer (IMS) operating near ambient temperature, ammonia-doped and equipped with a non-radioactive corona discharge ionization source. The instrument, model LCD-3.2E (Smiths Detection Ltd.), is based on a twin drift–cell time-of-flight configuration and simultaneously records ion mobility spectra in both positive and negative modes. DMNB generated distinct product ion peaks in both modes, with reduced mobility values (K_0_) of 1.42 cm^2^V^−1^s^−1^ (positive) and 1.37 cm^2^V^−1^s^−1^ (negative). The method demonstrated high sensitivity, with limits of detection calculated at 1.4 ppb_v_ (10.2 × 10^−3^ mg m^−3^) in positive mode and 3.1 ppb_v_ (22.7 × 10^−3^ mg m^−3^) in negative mode. The IMS system provided rapid responses within seconds and covered a quantifiable concentration range of 5–3000 ppb_v_, with saturation estimated to appear above approximately 5 ppm_v_ (36.6 mg m^−3^). The simultaneous dual-polarity response of the DT IMS enhances both the selectivity and reliability of identification. These findings confirm the capability of portable IMSs for fast trace vapor detection in DMNB, supporting its application in field-based screening scenarios such as luggage inspection or container interrogation, where indirect detection of plastic explosives is required.

## 1. Introduction

Trace detection of explosives remains a critical analytical challenge, particularly in the context of terrorist threats and transportation security. Detection approaches are typically divided into two large categories—bulk techniques (e.g., X-ray, neutron activation, millimeter-wave imaging) on one hand, and trace detection methods, which target minute quantities of explosive-related chemicals, on the other. While both have merits, trace detection offers key advantages: a high throughput, lower cost, and minimal invasiveness—a factor that strongly influences public acceptance.

Trace detection of explosives may imply either (a) direct detection and identification of explosive chemicals, or (b) indirect detection of explosives by detecting and identifying the so-called “chemical markers” or “chemical taggants”, which are much more volatile than the explosives themselves. Plastic explosives that contain RDX (1,3,5-trinitro-1,3,5-triazine, C_3_H_6_N_6_O_6_; known also as Cyclonite, Hexogen, or Royal Demolition eXplosive) or HMX (1,3,5,7-tetranitro-tetraazocyclooctane, C_4_H_8_N_8_O_8_; known as Octogen, or High Melting eXplosive) have extremely low vapor pressures; for instance, the vapor pressure of pure RDX is only 10^−9^ Torr at 20 °C, hence a volatility of ca. 30 ppt_v_; this volatility is in reality even lower because of the complex elastomeric matrix of a plastic explosive [1]. Therefore, direct detection of plastic explosives (like C-4) as vapors is practically almost impossible, and consequently trace detectors must vaporize solid particles, which were previously sampled by either swabbing or vacuuming people or their belongings.

To overcome this issue, the ICAO (International Civil Aviation Organization) came up with the solution of marking plastic explosives with more volatile compounds; it appears so far that the most suitable marker would be 2,3-dimethyl-2,3-dinitrobutane (DMNB), because it has a significant vapor pressure (ca. 2 × 10^−3^ Torr at 25 °C) and it does not seem to have any practical uses [2]. The level of the marker DMNB in plastic explosives as per the ICAO is at minimum 0.1% (*w*/*w*) [2], but usually 1% (*w*/*w*) of marking is employed by manufacturers of plastic explosives [3].

Various analytical technologies have been used for direct trace detection of explosives, from the golden standard of GC-MS to ion mobility spectrometry (IMS) [4]. Currently, it seems that IMS is the analytical technique for direct explosives detection used the most in the field, with more than 15,000 units deployed to all major airports and ports worldwide [5]. But again, marking plastic explosives during their production process, by the introduction of unique volatile compounds like DMNB into their matrix, will dramatically increase the probability of direct DMNB vapor detection and will also greatly simplify the sampling process, since no volatilization of solid particles will be needed.

The rapid detection of vapors emitted by volatile markers, such as DMNB, therefore has significant potential practical applications in identifying plastic explosives concealed in containers, luggage, and parcels. This method not only simplifies the detection of explosives but also contributes to enhancing security and border control, thereby having a significant positive impact on societal safety.

The principal objective of this work was to study and characterize the analytical responses generated by DMNB vapors using time-of-flight ion mobility spectrometry (IMS), near ambient temperature (approximately 25 °C) and with a non-radioactive ionization source. Specifically, the aim was to investigate the quantitative IMS response for DMNB, in both positive and negative operation modes.

Ion mobility spectrometry (IMS) is a fast and performant analytical technique, widely used for separating and identifying trace levels of vapors of substances present in air samples, after their soft ionization at atmospheric pressure [5]. The IMS technique has an exceptional sensitivity, since it successfully senses ionic currents in the range of pico-amperes (10^−12^ A). Rapid ion separation in the gaseous phase and at atmospheric pressure is most probably the main advantage of IMS, given the fact that a single spectrum is acquired in just twenty milliseconds; its outstanding sensitivity (low-ppb_v_ range for many classes of chemicals, without pre-concentration) would be the second paramount advantage. Also, the ruggedness, compactness and simple operation of IMS instrumentation have all led to the well-deserved, increasing popularity of IMS instruments, which are successfully used for a wide range of applications—from the rapid detection of explosives [6,7], illegal drugs [8,9] or chemical weapons and toxic industrial chemicals [10,11,12] to a continuously growing number of bio-medical [13], toxicological [14], industrial and agricultural [15,16] applications. Another very challenging application of IMS concerns detecting and discriminating between various microorganism strains [17,18]. IMS technology has also been employed in many other domains, like forensic analysis, industrial hygiene, biology, space research and investigation into the environment [5].

In Drift Tube (DT) IMS, ion separation takes place because they have different mobilities through a neutral drift gas (in portable instruments, purified air is usually employed) under the moving force exerted by the longitudinal D.C. electric field. Ionization in IMS is both gentle (meaning that the target analyte’s molecule is not fragmented) and occurs in two stages. Thus, if a radioactive ionization source is used with air as the drift gas, very fast and complex ion–molecule reactions lead to the formation of many ion clusters, called reactant ions; in positive ion mode and with traces of water vapor, prevalent positive reactant ions will be cluster ions of type (H_2_O)_x_H^+^, with lower amounts of (H_2_O)_y_NH_4_^+^ and (H_2_O)_z_NO^+^, while the predominant negative reactant ions are (H_2_O)_n_O_2_^−^. The positive or negative charge is then transferred from reactant ions to the analyte molecules; this way, the product ions are formed. Because the concentration of water vapor has a paramount role in atmospheric pressure ion–molecule chemistry, it must be carefully kept at a low and constant level inside the IMS measurement cell; otherwise, the analytical capabilities of the IMS instrument are going to degrade significantly [11]. When ammonia is used as a dopant, (H_2_O)_m_NH_4_^+^ clusters become the major positive reactant ions. Since the apparition of DT IMS in the 1970s, other variants of ion mobility-based techniques have emerged as well—like aspiration-type IMS (a-IMS) and differential mobility spectrometry (DMS) [5].

As a trace detection technology, DT IMS carries a series of consistent advantages over other ion mobility-based techniques such as aspiration IMS or differential mobility spectrometry. From this perspective, DT IMS is an already established and mature analytical tool, which was proven for crucial practical applications such as the fast detection of chemical warfare agents, illegal drugs or explosives. Also, DT IMS instrumentation is highly miniaturized, robust and pretty simple, thus its cost is often rather low, in the range of a thousand dollars per unit. The analytical output from a DT IMS device (ion current vs. drift time of ions) is very simple and readily understandable and interpretable, since it resembles a very fast chromatogram. In the case of an aspiration IMS, for instance, the analytical response is a set of ion currents produced by an array of multiple detectors, and is far more difficult to interpret. Besides its high integration, which resulted in handheld or even pocket-held commercial systems, DT IMS profits greatly from selectivity enhancement by altering the ionization chemistry through the use of so-called “dopants”; it is exactly the case for the miniaturized commercial instrument used in this study, which employs ammonia as a dopant for positive ion mode.

Ion mobility (noted as *K*) links the drift speed (*v_d_*) of a specific ion that travels inside the IMS cell to D.C. electric field intensity (*E*) that propels the ion towards the detector as follows: *v_d_* = *K* × *E* = *l_d_*/*t_d_*, where *l_d_* is drift length and *t_d_* is the drift time. Further, one may easily extract the ion mobility, as *K* = *v_d_*/*E* = *l_d_*/(*E* × *t_d_*). By normalizing *K* towards temperature and pressure, reduced ion mobility *K*_0_ is obtained: *K*_0_ = *K* × (*T_ambient_*/*T_cell_*) × (*P_cell_*/*P_atmospheric_*). *K*_0_ is the value used currently in IMS as a qualitative parameter, in order to both compare experimental results generated by various IMS instruments using diverse instrumental conditions and also to characterize a certain substance [5].

This study investigates the rapid detection and quantification of DMNB vapors using a handheld DT IMS device with corona discharge ionization, operating near ambient temperature. Both positive and negative ion modes were evaluated, and analytical performance parameters such as sensitivity, drift time, and detection limits were determined to assess the potential in real-world fast screening for plastic explosives.

## 2. Materials and Methods

**Chemicals and reagents**

Solid DMNB (2,3-dimethyl-2,3-dinitrobutane; C_6_H_12_(NO_2_)_2_; CAS No. 3964-18-9; white crystalline powder) with 98% purity was purchased from Sigma-Aldrich (St. Louis, MO, USA) and was used without purification. DMNB has a molecular weight M = 176.17 g mol^−1^, and therefore 1 ppm_v_ = 7.32 mg m^−3^ at 20 °C. Vapor pressure of DMNB is ca. 2 × 10^−3^ Torr at 25 °C. Apart from being used as a volatile chemical marker (taggant) for plastic explosives, DMNB has no known major industrial or commercial utilization.

**Vapor generation**

In order to perform the IMS measurements, a series of standard atmospheres with known low levels of DMNB vapors were generated by using dynamic methods. Thus, we employed a set of vapor sources, as follows: (a) a vapor source using permeation of DMNB vapors through a small plastic box made of polyethylene (PE), having a wall thickness of ca. 1.0 mm and total area of ca. 40 cm^2^, which was kept thermostated at ca. 37 °C, or (b) a vapor source that employed solely evaporation of solid DMNB introduced inside a glass vial with an internal volume of 20 cm^3^ and kept at a constant temperature of ca. 45 °C. Both vapor sources have used ca. 1 g of pure solid DMNB and were calibrated gravimetrically over a period of about two months using a laboratory ultra-microbalance model Sartorius ME-235S with a resolution of 10^−5^ g. Based on gravimetric measurements, we noticed an emission (permeation and evaporation, respectively) rate that varied by just ca. +/−10%.

A first set of test (standard) atmospheres with ultra-trace levels of DMNB vapors (of 5 ppb_v_ (0.035 mg m^−3^); 7 ppb_v_ (0.050 mg m^−3^); 10 ppb_v_ (0.075 mg m^−3^); and 20 ppb_v_ (0.150 mg m^−3^)) was generated by employing the first vapor source (a), which was based on permeation and had a very low permeation rate of R = 150 ng min^−1^. Air was utilized as dilution gas, after previous filtration by a combined scrubber with a mixture of molecular sieve 13X and activated charcoal; an electric air pump produced the diluting air flow; the flowrate was changed from 500 cm^3^ min^−1^ to 4500 cm^3^ min^−1^ by modifying the supply voltage and controlled using a rotameter calibrated for air and having a measuring range from 500 to 5000 cm^3^ min^−1^.

Using the vapor source (b) that was based solely on evaporation and having an evaporation rate R= 10,700 ng min^−1^, a second set of test atmospheres was generated, with known low concentrations of DMNB vapors: 300 ppb_v_, 600 ppb_v_, 1200 ppb_v_, and 3000 ppb_v_.

Exit gas flow rates for the standard atmospheres ranged therefore from 500 to 4500 cm^3^ min^−1^, for both vapor sources.

In conclusion, both concentration ranges were dictated by (a) the emission rates of the 2 vapor sources built and gravimetrically calibrated, and (b) the dilution air flowrates that were employed (from 500 to 4500 cm^3^ min^−1^). The two ranges were further utilized in order to investigate the DT IMS instrument detection capability in both low-ppb_v_ domain (ultra-trace levels) and in the ppm_v_ domain (trace levels).

**Instrumentation**

For all investigations on the detection and quantification of the target analyte (DMNB vapors) the pocket-held DT IMS instrument model Lightweight Chemical Detector LCD-3.2E, produced by Smiths Detection Ltd., Watford, UK, was used. This portable IMS instrument has a classic design cell with stacked rings, with discrete metallic rings placed on the insulating cylinder of the measuring cell; the drift length *l_d_* of the miniature IMS cell is approximately 30 mm and the electric field applied onto it has an intensity of ca. 270 V cm^−1^. The measuring cell is operated near ambient temperature, at ca. 25 °C, and the pressure inside is atmospheric pressure. Ionization source is a non-radioactive one—a corona discharge. As neutral drift gas is utilized dry air, which is recirculated using a closed-loop, internal pneumatic system’s main element is a filter with 10 A molecular sieve. The filter material delivers, in a constant manner, controlled low levels of ammonia, which is a dopant that, by changing ionization chemistry at atmospheric pressure, enhances selectivity in positive ion mode. The Lightweight Chemical Detector model LCD-3.2E is currently one of the smallest commercial DT IMS instruments on the market, being especially designed to rapidly detect vapors of both toxic industrial chemicals and chemical warfare agents. The LCD-3.2E instrument has the particular feature of employing two miniature drift cells that are mounted next to each other (a so-called “twin IMS cell”); this particular design generates both positive and negative ion mobility spectra at the same time. This compact commercial IMS instrument has been described in detail in ref. [12], including its principle and schematic diagram. Air samples were drawn inside the LCD-3.2E by using the analytical/survey nozzle, and not its standard rain-protecting cap. The IMS instrument was connected to a PC computer and operated using the proprietary IMS control software TrimScan2, ver. 0.4.0 (Smiths Detection Ltd.). Analytical data were also saved on the hard disc as ion mobility spectra (positive and negative), then they were exported as MS Excel files using the same proprietary software, TrimScan2.

## 3. Results

### 3.1. Experimental Results

Experimental data generated as ion mobility spectra by the DT IMS instrument model LCD-3.2E (ammonia-doped and operated near ambient temperature) were recorded for each of the seven concentration levels of DMNB vapors in test atmospheres. The IMS experiments were run in triplicate for each concentration of DMNB vapors and standard deviations between 2% and 7% were noticed. The results are summarized in Table 1, where C_DMNB_ is the concentration of DMNB in the corresponding test/standard atmosphere.

DMNB produced an IMS response in both positive and negative ion modes, simultaneously. The ion mobility spectrometric response consisted of characteristic spectra, where one distinct product ion peak (PIP) was observed in the positive ion mode, at drift time t_d_ = ca. 7.5 ms, while in the negative operation mode one clearly separated PIP was noticed at t_d_ = ca. 7.6 ms. The positive reactant ion peak (POS RIP) was observed at a drift time t_d_ = ca. 4.6 ms and negative reactant ion peak appeared at t_d_ = ca. 4.8 ms.

**Figure 1:**
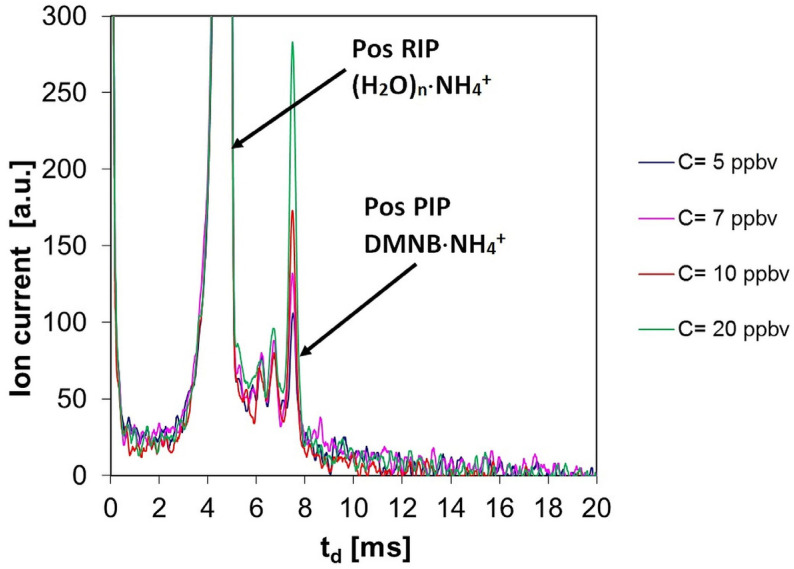
Ion mobility spectra from DMNB, obtained in the positive ion mode—for very low vapor concentrations—ranging from 5 to 20 ppb_v_.

Ion mobility spectra obtained in the positive mode for the very low concentrations (from 5 to 20 ppb_v_ DMNB) are shown in Figure 1; in order to better visualize the positive product ion peak, the Y axis was “zoomed” to 300 a.u.

**Figure 2:**
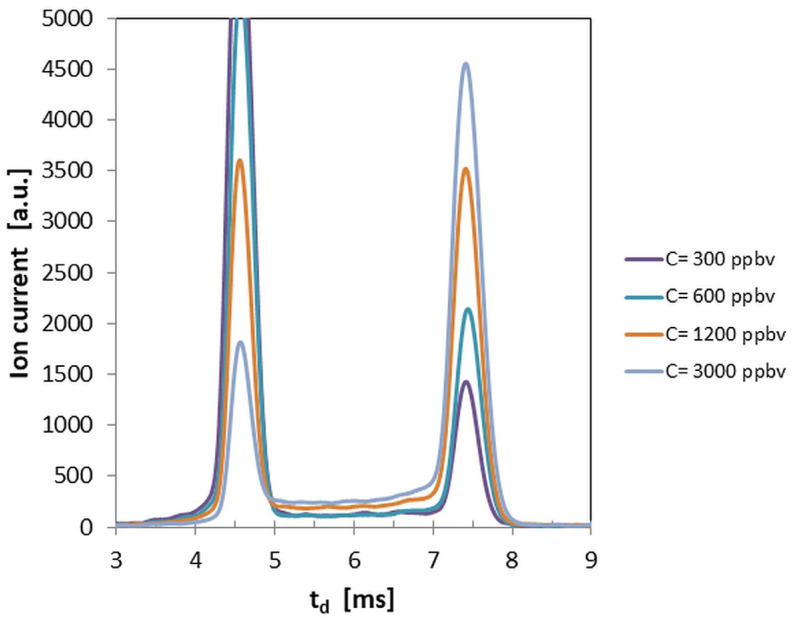
Ion mobility spectra from DMNB vapors, obtained in the positive ion mode for low vapor concentrations (from 300 to 3000 ppb_v_). The peak at the left (t_d_ ca. 4.6 ms) is the positive reactant ions’ peak, while the peak at the right (t_d_ ca. 7.4 ms) is the positive product ion peak generated by DMNB.

Further, in Figure 2, ion mobility spectra obtained in the positive ion mode for low concentrations of DMNB vapors—from 300 to 3000 ppb_v_—are shown. These spectra illustrate how the conservation of electrical charge occurs: when the vapor concentration of DMNB increases, the generation of product ions (that embed the molecule of the analyte) takes place by transferring the positive charge from positive reactant ions. This way, by increasing the peak height of the positive product ion peak of DMNB, the intensity of the positive reactant ion peak diminishes.

The positive spectrum presented in Figure 2 at 3000 ppb_v_ DMNB still shows a detectable positive reactant ion peak (RIP). Its intensity is approximately 1700 a.u., while the RIP intensity in the blank spectrum is about 8000 a.u., corresponding to roughly 20% of the initial signal. Based on this observation, we interpreted that complete saturation had not yet occurred within the investigated concentration range.

**Figure 3:**
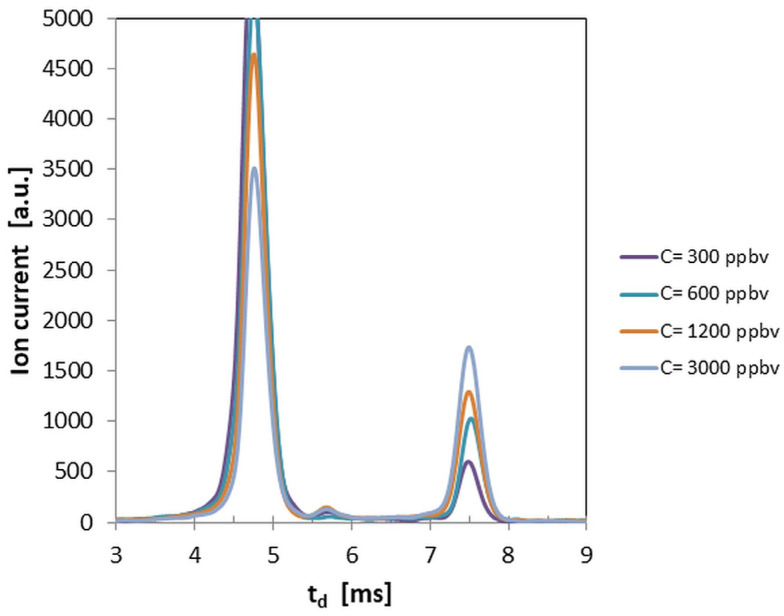
Ion mobility spectra from DMNB vapors, obtained in the negative ion mode for low vapor concentrations (from 300 to 3000 ppb_v_). The peak at the left is the negative reactant ions’ peak (t_d_ ca. 4.8 ms), while the peak at the right (t_d_ ca. 7.6 ms) is the negative product ion peak generated by DMNB.

Ion mobility spectra obtained in the negative mode for low concentrations (from 300 to 3000 ppb_v_ DMNB) are shown in Figure 3.

**Figure 4:**
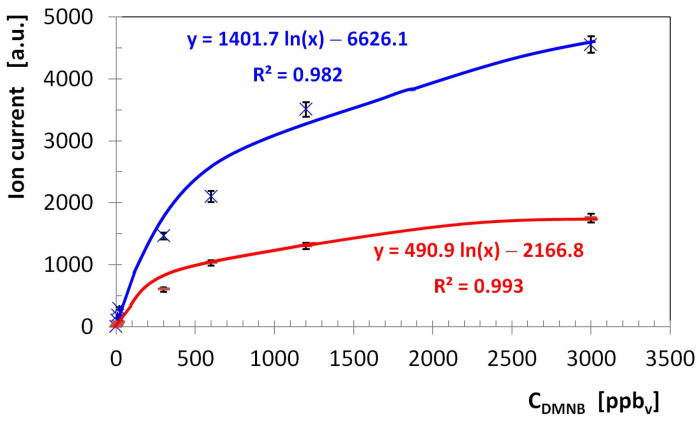
Calibration for DMNB: blue crosses and line for positive ion mode and red dashes and line for negative ion mode. Error bars show the standard deviation of three repetitive measurements (n = 3). Each calibration curve comprises two portions—a linear one for ultra-trace levels of DMNB (from low ppb_v_ to ca. 100 ppb_v_, with regression equations and R^2^ provided in the validation sub-section), followed by a logarithmic one for medium and high concentrations of DMNB vapors (higher than ca. 100 ppb_v_). Equations of logarithmic regressions and R^2^ are included in this figure.

In order to assess the quantitative response of the LCD-3.2E pocket-held IMS instrument to vapors of DMNB, quantitative data were plotted and the calibration curve was generated for both ion polarities. The calibration curve is presented in Figure 4; the signal (ion current) represents the peak height for product ion. Calibration curve data points were calculated as the mean of three independent measurements. The relative deviation ranged from 3 to 8% in the positive ion mode and from 4 to 9% in the negative ion mode.

Qualitative information associated with IMS spectra is related to the drift time *t_d_* of an ion, which determines the reduced mobility *K*_0_ of that ion, while quantitative information is linked to the area (or peak height) of product ions’ peaks. In a nutshell, any ion mobility peak that appears in the spectrum generated by the LCD-3.2E instrument can be described synthetically by using just three essential numbers: (1) drift time *t_d_* (in ms); (2) reduced mobility *K*_0_ (in cm^2^V^−1^s^−1^); and (3) peak height *h_max_* (in a.u.).

Qualitative information is displayed in Table 2 and basically refers to the identification of the target analyte on the basis of reduced ion mobility *K*_0_ for DMNB product ion peaks (in both positive ion mode and negative ion mode).

Besides the K_0_ value provided by the IMS instrument software TrimScan, we have also calculated reduced ion mobility K_0_ by using the so-called “IMS cell constant”; this methodology has the advantage of taking into account, in a comprehensive manner, all the factors that could potentially influence drift time and K_0_, for instance the effects due to minor non-homogeneities in the electric field E that moves the ions along the IMS cell. Using the IMS cell constant also excludes the necessity of accurately measuring the instrumental parameters (such drift length *l_d_* and intensity of electric field *E*) and environmental parameters (like temperature and pressure inside the measurement cell). Methyl salicylate (MSAL) has already been extensively used as a chemical standard in the negative operation mode of IMS devices, having an established reduced ion mobility K_0 of standard (MSAL)_ = 1.474 cm^2^V^−1^s^−1^ [11], while in the positive ion mode 2,4-lutidine (2,4-dimethylpyridine) is widely accepted as a chemical standard, with a reduced ion mobility K_0 of standard (Lutidine monomer)_ = 1.950 cm^2^V^−1^s^−1^ and K_0 of standard (Lutidine dimer)_ = 1.430 cm^2^V^−1^s^−1^ [19,20]. The ion mobility cell constant (noted here by A) in fact represents the product between K_0_ and the drift time of the chemical standard product ion. In other words, in order to calculate the reduced mobility of DMNB’s product ion peak in the positive ion mode, the following formula is used: A = K_0 of standard (Lutidine dimer)_ × t_d of standard (Lutidine dimer)_ = K_0 of analyte (DMNB)_ × t_d of analyte (DMNB)_. Since t_d of standard (Lutidine dimer)_ = 7.44 ms, then the cell constant will be: A = 1.43 cm^2^V^−1^s^−1^ × 7.44 × 10^−3^ s = 10.639·10^−3^ cm^2^ V^−1^. In a similar manner, using methyl salicylate MSAL as a chemical standard in the negative ion mode (with t_d_ = 7.02 ms), the cell constant will be: A = 1.474 cm^2^V^−1^s^−1^ × 7.2 × 10^−3^ s = 10.348·10^−3^ cm^2^ V^−1^.

The ratio between the drift times of PIP and RIP (equivalent to the ratio of reduced mobilities), is: t_d Pos PIP_/t_d Pos RIP_ = K_0 Pos RIP_/K_0 Pos PIP_ = 1.626 for the product ion in the positive ion mode (assignable to DMNB·NH_4_^+^), and t_d Neg PIP_/t_d Neg RIP_ = K_0 Neg RIP_/K_0 Neg PIP_ = 1.573 for the product ion in the negative ion mode (assignable to DMNB·O_2_^−^), respectively. The above-mentioned ratio actually normalizes product ions’ drift time (and associated *K*_0_) against the drift time (and reduced ion mobility) of the reactant ion peak; in other words, the reactant ions’ peak is taken as the benchmark.

For an IMS instrument, resolving power is defined as the ratio between the drift time of a certain ion and the width at half height (*R_IMS_* = *t_d_*/Δ*t_d_*) and was calculated for all the peaks present in the spectra. The obtained results are presented in Table 3.

Our obtained values for resolving power are normal for commercial IMS instruments that are equipped with miniaturized cells, like the case of the pocket-held LCD-3.2E instrument used.

### 3.2. Validation

In order to evaluate the suitability of the analytical method, a rapid validation process was performed. The parameters assessed were: the limit of detection, limit of quantitation, sensitivity, and accuracy of the proposed method using IMS.

Both the limit of detection LoD and limit of quantitation LoQ were derived, as outlined by IUPAC, from the parameters of the calibration line (namely, the standard deviation of the response and the slope). Sensitivity S is calculated as the modification of signal Y (in this case, peak height) that occurs when the concentration is changed (S = ΔY/ΔC). The figures of merit concerning DMNB detection are summarized in Table 4; they were calculated using the ultra-trace levels of DMNB vapors, from 5 to 20 ppb_v_.

Precision was evaluated using analyses in triplicate (see Table 1). Accuracy was assessed by using the relative standard deviation RSD (also called the coefficient of variation CV), which was between 2% and 7% for the PIP in both positive and negative ion modes. Good repeatability of the results was noticed, with RSD < 10%.

## 4. Discussion

We showed that, under the described experimental conditions (near ambient temperature), DMNB simultaneously generated both positive and negative IMS spectra; these spectra are simple, with only one product ion peak that is well separated from the reactant ion peak. This feature dramatically increases the confidence of detection for DMNB vapors by IMS, since a lower number of compounds generate ion mobility spectra in the negative ion mode, compared to the number of compounds that produce spectra in the positive ion mode; moreover, even fewer chemicals simultaneously generate positive and negative ion mobility spectra.

Since vapors of DMNB have elicited an IMS response in the positive ion mode, it is evident that DMNB possesses a proton affinity (PA) higher than the PA of the ammonia dopant used in the LCD-3.2E instrument. The pocket-held instrument model LCD-3.2E has also provided a very fast response, in real time (several seconds), to vapors of DMNB.

The definite identification and characterization of ions existing inside the IMS measurement cell (either reactant or product ions) can be performed only by using complex hyphenated IMS-MS systems and was not in fact the purpose of this study. However, other authors found that in the IMS systems with ammonia used as a dopant and operated at temperatures below 50 °C (conditions that are fully satisfied in the IMS instrument model LCD-3.2E we used), the product ions generated by OP compounds were in fact ammoniated clusters. For example, the assignation of product ions from DMMP (a surrogate molecule for nerve agent Sarin) as DMMP·NH_4_^+^ and DMMP_2_·NH_4_^+^ has already been done using mass spectrometry by Hauck et al. [21].

We succeeded in avoiding reaching saturation in the IMS instrument, since even at our highest vapor concentration investigated, ca. 3 ppm_v_ vapors of DMNB, saturation did not appear in the positive ion mode—a fact demonstrated by the survival of ca. 20% of the initial positive reactant ions’ peak. Because at saturation the full amount of reactant ions were consumed, the visible effect in the IMS spectrum is the disappearance of the reactant ions’ peak. In any case, the saturation of an ion mobility spectrometric cell definitely has to be avoided, because it will most probably induce a long-term contamination of both the measurement cell and any internal surfaces that come in contact with the analyte; negative memory effects and associated false alarms follow.

Potential chemical interferences may always become a problem, and this is of course valid for IMS instruments as well. However, because DMNB vapors simultaneously produce product ion peaks in both positive and negative polarities, the identification of DMNB while simultaneously using the two time windows associated with those two mentioned product ion peaks that appear in both polarities adds a lot more certainty to the detection and identification, compared to those based on a single product ion peak and/or using just one polarity. We consider that there is a very low probability, in a real-world screening scenario, that a common environmental contaminant or some chemicals from personal care products would generate a perfectly similar IMS response as that of the DMNB marker—a simultaneous dual-polarity IMS response, with only one well-resolved product ion peak having a reduced ion mobility K_0_ of ca. 1.40 cm^2^V^−1^s^−1^ in both positive and negative ion modes and showing a positive-to-negative intensity ratio of about 2:1. As already pointed out, there are very few compounds forming in DT IMS both positive and negative product ions simultaneously.

Figures of merit given in the scientific literature for DMNB detection and quantification by various IMS instruments, and those found by us, are also summarized in Table 5, where LoD is the limit of detection and LoQ the limit of quantification.

Reduced ion mobilities for the positive and negative product ion peaks have been found to be K_0_ = 1.42 cm^2^V^−1^s^−1^ (positive ion peak, which could probably be assigned to DMNB·NH_4_^+^ [24]) and K_0_ = 1.37 cm^2^V^−1^s^−1^ (negative ion peak, which could probably be assigned to DMNB·NO_2_^−^ [24]). Both values of K_0_ are in good accordance with other results found in the literature (see Table 5).

Our study addressed the quantification of vapors of DMNB volatile taggant, in the vapor phase, using a pocket-held twin-cell IMS operated near ambient temperature. Therefore, our focus towards the real-time vapor quantification of the volatile DMNB marker using pocket-held IMS devices with a simultaneous dual-polarity response complements particle-based approaches (which are performed using mostly desktop or laboratory-based IMS systems), underlining both the originality and practical relevance of the present work.

One easily notices from Table 5 that the literature concerning DMNB detection by IMS is pretty scarce—as we have identified only six papers—and focused merely on qualitative or semi-quantitative aspects; also, most approaches so far have addressed IMS sensing of DMNB using only the positive ions. In other words, previous studies did not address, in a comprehensive manner, the quantitative aspects of DMNB vapor sensing in both ion polarities. In this study, we have provided a thorough quantitative insight for both positive and negative ion modes, using standard atmospheres created by continuous dynamic methods that were centered on gravimetrically calibrated vapor sources based on permeation and evaporation. This way, we were able to generate even test atmospheres with DMNB vapor concentrations in the low-ppb_v_ range, which allowed us in turn to obtain the afferent IMS spectra for DMNB levels at and near the LoQ (Figure 1); we believe that this consolidates the originality of our study. Moreover, we have demonstrated that the sensitivity in positive ion mode is about three times better than in negative ion mode. The bottom line is that the cornerstone of our approach is the preparation of the above-mentioned standard/test atmospheres with ultra-trace (ppb_v_) and trace (ppm_v_) levels of DMNB vapors.

The excellent paper by Ewing et al. [24] investigated the detection of two volatile markers for plastic explosives, namely ethyleneglycol dinitrate (EGDN) and 2,3-dimethyl-2,3-dinitrobutane (DMNB), using a handheld ion mobility spectrometer model LCD. However, their findings did not cover the quantification of DMNB, since no LoD/LoQ was indicated and a calibration curve for this taggant was not provided; the vapor source used by them relied on the slow evaporation of 0.5 mg DMNB that was deposited, as a solution of 1 mg cm^−3^ DMNB in either acetone or methanol, onto 1 g of molecular sieve 13X.

The examination of the quantitative response (see Figure 4) and of the ion mobility spectra obtained for all the investigated concentration levels of DMNB vapors (see Figure 1, Figure 2 and Figure 3) allows us to infer that:

1. Limits of detection are in the low ppb_v_ range—1.4 ppb_v_ (10.2 µg m^−3^) in positive ion mode, and 3.1 ppb_v_ (22.7 µg m^−3^) in negative ion mode.
2. The minimum measured concentration of DMNB vapors was just 5 ppb_v_ (36.6 µg m^−3^) in positive ion mode, and 20 ppb_v_ (146.4 µg m^−3^) in negative ion mode. In other words, we succeeded in performing real measurements close to the calculated LoQs.
3. The sensitivity of detection in positive ion mode is about 2.25 times better than that in negative ion mode (see Figure 4 and Table 4).
4. Saturation is estimated to appear at levels above 5000 ppb_v_ (36.6 mg m^−3^) DMNB in positive ion mode. Of course, this figure indicates just a projection based on the observed trend, rather than a definitive experimental finding.

Looking at limits of detection and limits of quantification for vapors of DMNB using a pocket-held IMS instrument with corona discharge ionization and operated near ambient temperature, model LCD-3.2E, in the positive ion mode these LoDs and LoQs are just about one order of magnitude poorer than those obtained for organophosphorus (OP) compounds. For instance, the LoD and LoQ for DMNB in the positive ion mode (1.4 and 4.2 ppb_v_, respectively) were found to be only about five times poorer than those obtained, using exactly the same IMS instrument and under similar conditions, for di-isopropyl methyl phosphonate DIMP (0.24 and 0.80 ppb_v_), which is a known surrogate compound for nerve chemical warfare agents [28].

## 5. Conclusions

The focus of this research was to determine the feasibility of using the pocket-held IMS instrument (model LCD 3.2E) to detect vapors of the DMNB explosive marker present in air samples and also to determine detection and quantification limits when operating near ambient temperatures.

The fast detection and characterization of explosive markers by IMS, including DMNB, is not new. Although DMNB has already been investigated using IMS, most research has focused on the qualitative or semi-quantitative detection of DMNB. This work’s main goal was therefore to get comprehensive information regarding the figures of merit related to DMNB quantification using a pocket-held portable ion mobility spectrometer with corona discharge ionization operated near ambient temperature, model LCD-3.2E (Smiths Detection, Ltd.).

Vapors of DMNB were quickly detected and quantified, in real-time (several seconds), both in positive and negative ion modes. Simple ion mobility spectra were obtained, containing just one product ion peak that was well separated from the reactant ion peak. Both reduced ion mobilities—for positive and negative product ions—had values close to 1.40 cm^2^V^−1^s^−1^ (K_0_ = 1.42 cm^2^V^−1^s^−1^ for the positive product ion, and K_0_ = 1.37 cm^2^V^−1^s^−1^ for the negative product ion), which are in excellent agreement with those found by Ewing and Miller [24]. Consequently, the fast identification of DMNB may be reliably accomplished based simultaneously on these two product ions.

A seven-point calibration curve for DMNB vapors from 5 to 3000 ppb_v_ (0.037 to 22 mg m^−3^) was built. The detection limit was found to be LoD 1.4 ppb_v_ (0.010 mg m^−3^) in positive ion mode and 3.1 ppb_v_ (0.023 mg m^−3^) in negative ion mode, while the limit of quantification is LoQ 4.2 ppb_v_ (0.031 mg m^−3^) in positive ion mode and 9.4 ppb_v_ (0.069 mg m^−3^) in negative ion mode. Saturation of the IMS response is expected to appear at concentration levels above 5 ppm_v_ (36.6 mg m^−3^) of DMNB. The minimum measured concentration of DMNB was ca. 5 ppb_v_ in the positive ion mode. This information is in good agreement with the scarce information found in the literature.

Altering ionization chemistry at atmospheric pressure in the positive ion mode by using ammonia doping offers the essential advantage of preventing competitive ionization (hence chemical interference) from a large number of chemicals; this way, selectivity is greatly enhanced, since any chemical that possesses a PA smaller than the PA of ammonia (854 kJ mol^−1^) will not be able to take the positive charge from the ammonium-positive reactant ions.

Through this study, we have succeeded to prove that pocket-held, low-resolution DT IMS instrumentation equipped with a non-radioactive ionization source is absolutely adequate for quickly sensing and quantifying vapors of DMNB at ultra-trace levels (parts per billion in volume), while providing a series of valuable strategical advantages—of which the most relevant is the real-time response, in seconds—compared to other analytical techniques.

To the best of our knowledge, this work is the first that:

(a) Quantitatively explores the ultra-trace levels of DMNB vapors at ambient temperature using a pocket-held portable DT IMS system with corona ionization and ammonia-doped; ultra-trace levels of only 5 ppb_v_ of DMNB—by preparing standard atmospheres using a dynamic method based on permeation vapor sources—were measured in the positive ion mode, which is just around the calculated limit of quantification.
(b) Describes in detail the simultaneous IMS response—in both positive and negative ion mode, and establishes that the positive response is about two times stronger than the negative one. The IMS response for DMNB vapors was simple; it consisted, in both ion modes, of only one product ion peak, with reduced ion mobility K_0_ around the value of 1.40 cm^2^V^−1^s^−1^. The simultaneous presence of a positive and negative IMS response definitely represents an advantage in the qualitative identification of the target compound.
(c) Proves that IMS figures of merit for DMNB marker vapors are close to those obtained for some organophosphorus (OP) compounds; for instance, in positive ion mode the LoD and LoQ for DMNB were found to be less than one order of magnitude poorer than those determined for OP compounds.

This paper presents, to the best of our knowledge for the first time, the quantitative results of investigating trace vapors of DMNB, which is the main and most utilized volatile marker of plastic explosives, in both positive and negative ion modes, using a handheld DT IMS operated near ambient temperature. The quantitative characterization of DMNB vapors presented in this study opens the way for further research regarding, for instance, the amount of DMNB generated by a solid matrix that simulates a block of plastic explosive, which embeds the ICAO-regulated amount of DMNB volatile marker.

As a conclusion, the fast detection of DMNB as a chemical marker of plastic explosives in the field, in real time and at ultra-trace levels (low parts per billion, ppb_v_) is perfectly feasible by using simple, portable, lightweight IMS instruments, such as the pocket-held LCD-3.2E device. This way, the fast indirect detection of plastic explosives would be possible using highly portable, simple and compact analytical systems based on ion mobility spectrometry.

## Figures and Tables

**Table 1:** Summary of quantitative results obtained with DMNB vapors using the LCD-3.2E pocket-held DT IMS instrument in both positive ion mode and negative ion mode (three replicates were used for peak height, in order to calculate standard deviation).

C_DMNB_ [ppb_v_]	IMS Data—Positive Ion Mode		IMS Data—Negative Ion Mode	
	Drift Time t_d_ [ms]	Peak Height h_max_ [a.u.]	Drift Time t_d_ [ms]	Peak Height h_max_ [a.u.]
5	7.48	105 ± 7	7.58	30 ± 2
10		180 ± 11		50 ± 3
20		290 ± 15		75 ± 5
300		1450 ± 46		600 ± 28
600		2100 ± 72		1030 ± 36
1200		3500 ± 94		1300 ± 42
3000		4550 ± 107		1750 ± 57

**Table 2:** Reduced ion mobilities K_0_ for ions produced by DMNB (positive and negative ion mode).

Operation Mode	Ion Drift Time, t_d_ [ms]	Reduced Ion Mobility ^1^, K_0_ [cm^2^V^−1^s^−1^]	Reduced Ion Mobility ^2^, K_0_ [cm^2^V^−1^s^−1^]
Positive	Pos RIP: 4.60	2.330	2.314
	Pos PIP: 7.48	1.435	1.422
Negative	Neg RIP: 4.82	2.234	2.147
	Neg PIP: 7.58	1.414	1.365

^1^—Calculated by the IMS instrument software TrimScan. ^2^—Calculated using the IMS cell constant (A): K_0_ = (A/td).

**Table 3:** Resolving power of the LCD-3.2E IMS instrument for DMNB (positive and negative ion mode) at vapor concentration of 600 ppb_v_.

Ion Drift Time, t_d_ [ms]	Peak Width At Half Maximum, Δt_d_ [ms]	Resolving Power, R_IMS_
Pos RIP: 4.56	0.34	13.4
Pos PIP: 7.44	0.38	19.6
Neg RIP: 4.76	0.32	14.9
Neg PIP: 7.52	0.33	22.8

**Table 4:** Analytical figures of merit related to IMS detection of DMNB (for both positive and negative ion mode).

Ion Mode	LoD [ppb_v_]	LoQ [ppb_v_]	Equation	R^2^	S [a.u./ppb_v_]
Positive	1.4	4.2	Y = 11.75·X + 49.9	0.997	12
Negative	3.1	9.4	Y = 2.70·X + 20.9	0.987	3

**Table 5:** Previous work on DMNB sensing using various DT IMS systems. Legend: RS—instrument equipped with a radioactive ionization source; NRS—instrument equipped with a non-radioactive ionization source.

DT IMS Instrument & Experimental Conditions	K_0_ [cm^2^V^−1^s^−1^]	LoD	Reference
RS IMS of CAM type (4.25 cm drift cell) & IMS-MS Operating temperature: 318 K (45 °C). No decomposition of DMNB was observed. Several dopants were investigated: water, ammonia, and dichloromethane. Concentrations investigated: 0.23 mg m^−3^ (30 ppb_v_) and 1.2 mg m^−3^ (165 ppb_v_). Identity of reactant & product ions was assigned by IMS-MS. Only positive ion mode was explored.	1.43 … 1.47 (positive mode; 318K)	n/a	[22]
High-T IMS using a 4.25 cm long drift cell (RS). Operating temperature: between 317 and 455 K. Several dopants were investigated: water, ammonia, and dichloromethane. Concentrations investigated: 0.23 mg m^−3^ (30 ppb_v_) and 1.2 mg m^−3^ (165 ppb_v_). Ammonia doping was observed to produce the most sensitive and clearly resolved response to DMNB.	1.48 (positive mode; 455 K) Decomposition of DMNB was noticed at high T (>50 °C)	n/a	[23]
DT IMS with corona discharge ionization, ammonia doping and miniaturized drift cell, model LCD (NRS).	1.44 (positive mode) 1.33 (negative mode)	n/a	[24]
SPME + IMS (RS, model GE Itemiser 2), drift tube temp. 50 °C.	n/a	0.31 ng	[25]
SPME + IMS (RS, model GE Itemiser 2), drift tube temp. 50 °C. IMS (RS, model IonScan 400B).	1.40 (positive mode; water chemistry)	1.6 ng	[26]
SPME + IMS (RS, model GE Itemiser 2), drift tube temp. 50 °C.	1.40 (positive mode)	1.6 ng	[27]
Current study: DT IMS, model LCD-3.2E (NRS); dopant: NH_3_ Drift tube temp. ca. 25 °C. Calibration: from 5 to 3000 ppb_v_ Linear range: from 5 to ca. 100 ppb_v_ (estd.) Saturation: >5000 ppb_v_.	1.42 (positive mode) 1.37 (negative mode)	LoD 1.4 ppb_v_ (Pos mode) LoD 3.1 ppb_v_ (Neg mode)	

n/a—not available.

## Data Availability

The original contributions presented in the study are included in the article; further inquiries can be directed to the corresponding authors.

## Abbreviations

The following abbreviations are used in this manuscript:

DMNB	2,3-dimethyl-2,3-dinitrobutane
ICAO	International Civil Aviation Organization
DT IMS	Drift tube ion mobility spectrometry
RIP	Reactant ion peak
PIP	Product ion peak
PA	Proton affinity
OP	Organo-phosphorus
DMMP	Dimethyl methylphosphonate
DIMP	Diisopropyl methylphosphonate
